# Supplementary material for: Inhibiting Fatty Acid Oxidation Suppresses Acquired Resistance to Standard Chemotherapy in Melanoma
Source: Int J Mol Sci. 2025 Oct 10;26(20):9873. doi: 10.3390/ijms26209873 (PMC12563080; doi:10.3390/ijms26209873)
Supplement: Supplementary file 1 [file ijms-26-09873-s001.zip › ijms-3916413-supplementary.pdf]

Supplementary Figure S1.

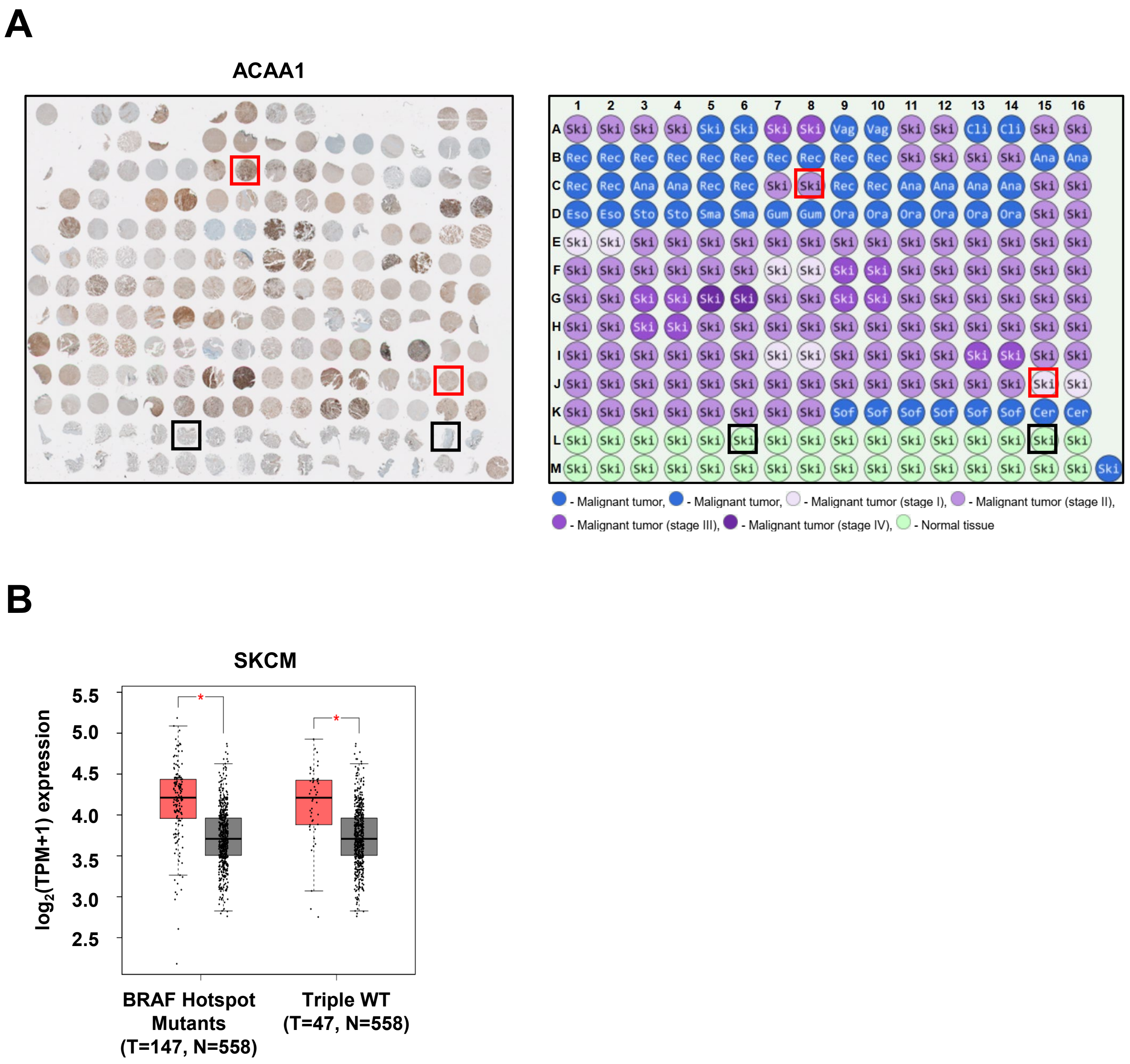

Supplementary Figure S1. Expression of FAO enzyme in melanoma patients. (A) Immunohistochemical staining of melanoma tissue microarray (TMA; ME2081, Tissue Array, Derwood, MD, USA) using an anti-ACAA1 antibody. **(B)** Expression levels of eight fatty acid oxidation (FAO)-related genes — CAC (carnitine acyl-carnitine carrier), CPT1A (carnitine palmitoyltransferase 1A), ACAA1 (acetyl-Coenzyme A acyltransferase 1), ACAA2 (acetyl-Coenzyme A acyltransferase 2), EHHADH (enoyl-CoA hydratase and 3-hydroxyacyl CoA dehydrogenase), ACADM (acyl-CoA dehydrogenase medium chain), ACOT4 (acyl-CoA thioesterase 4), and HADHB (hydroxyacyl-CoA dehydrogenase trifunctional multienzyme complex subunit beta) — in BRAF-mutant and wild-type melanoma compared with matched normal tissues. Data were obtained from TCGA and GTEx datasets using the GEPIA2 "Expression DIY" module with the following parameters: $\log_2$ fold change cutoff = 0.5 and p-value cutoff = 0.05. T = tumor (red); N = normal (grey).

Supplementary Figure S2.

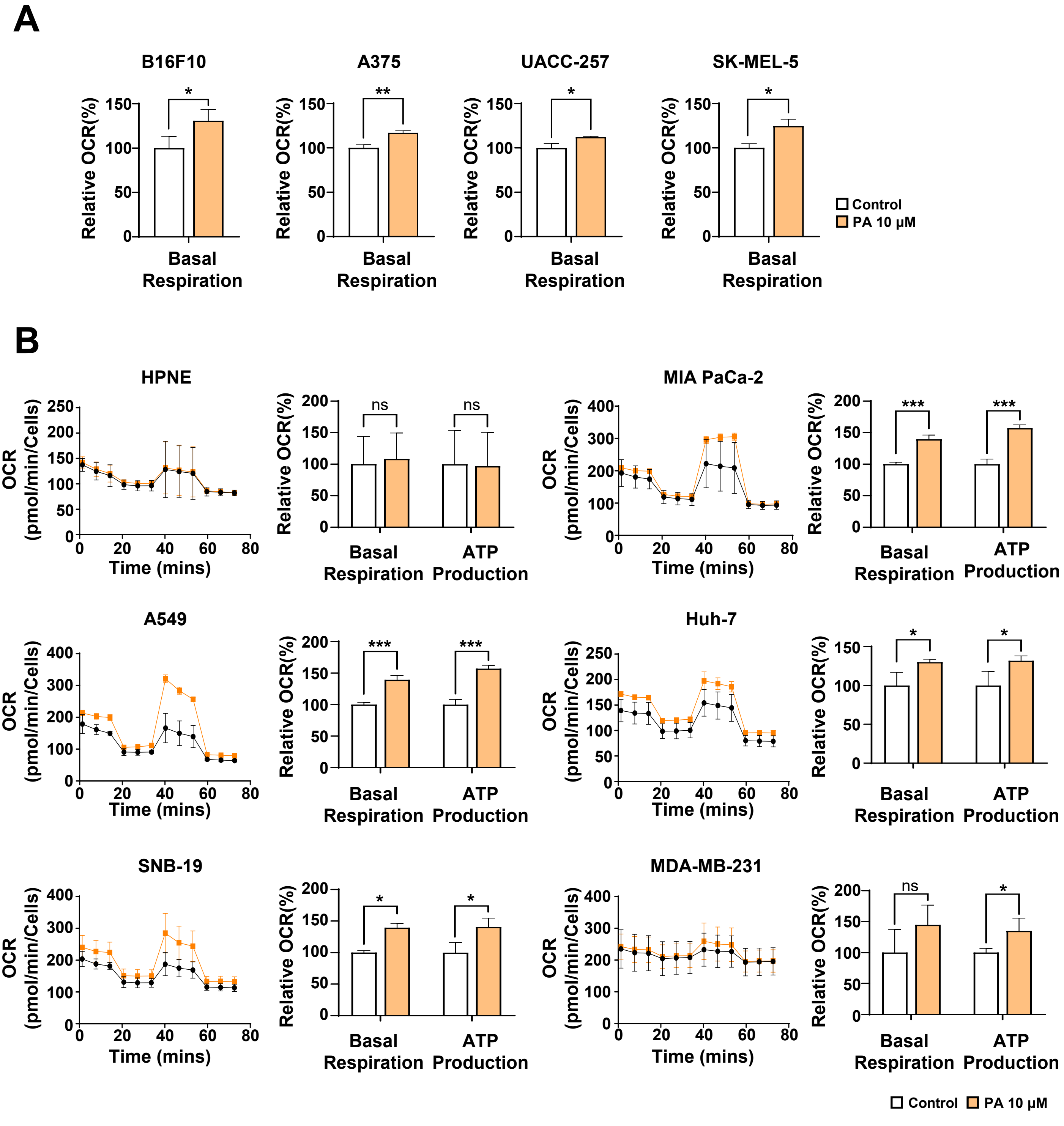

Supplementary Figure S2. Basal respiration and ATP production were increased upon palmitic acid treatment in various cancer cells. Oxygen consumption rate (OCR) analysis was performed using the Seahorse XFe96 analyzer to determine basal respiration and ATP production. All data were normalized to SRB assay quantification. **(A)** B16F10, A375, UACC-257, and SK-MEL-5 cells were treated with BSA-conjugated palmitic acid (PA, 10 μM) for 3 h. Basal respiration increased in the fatty acid-treated groups compared with controls. **(B)** HPNE, MIA PaCa-2, A549, Huh-7, SNB-19, and MDA-MB-231 cells were treated with BSA-conjugated palmitic acid (PA, 10 μM) for 3 h. All cancer cell lines tested, but not the normal pancreatic cell line HPNE, showed increased basal respiration and ATP production following fatty acid treatment. * $p < 0.05$, ***$p < 0.001$ vs. Control; ns, not significant.

Supplementary Figure S3.

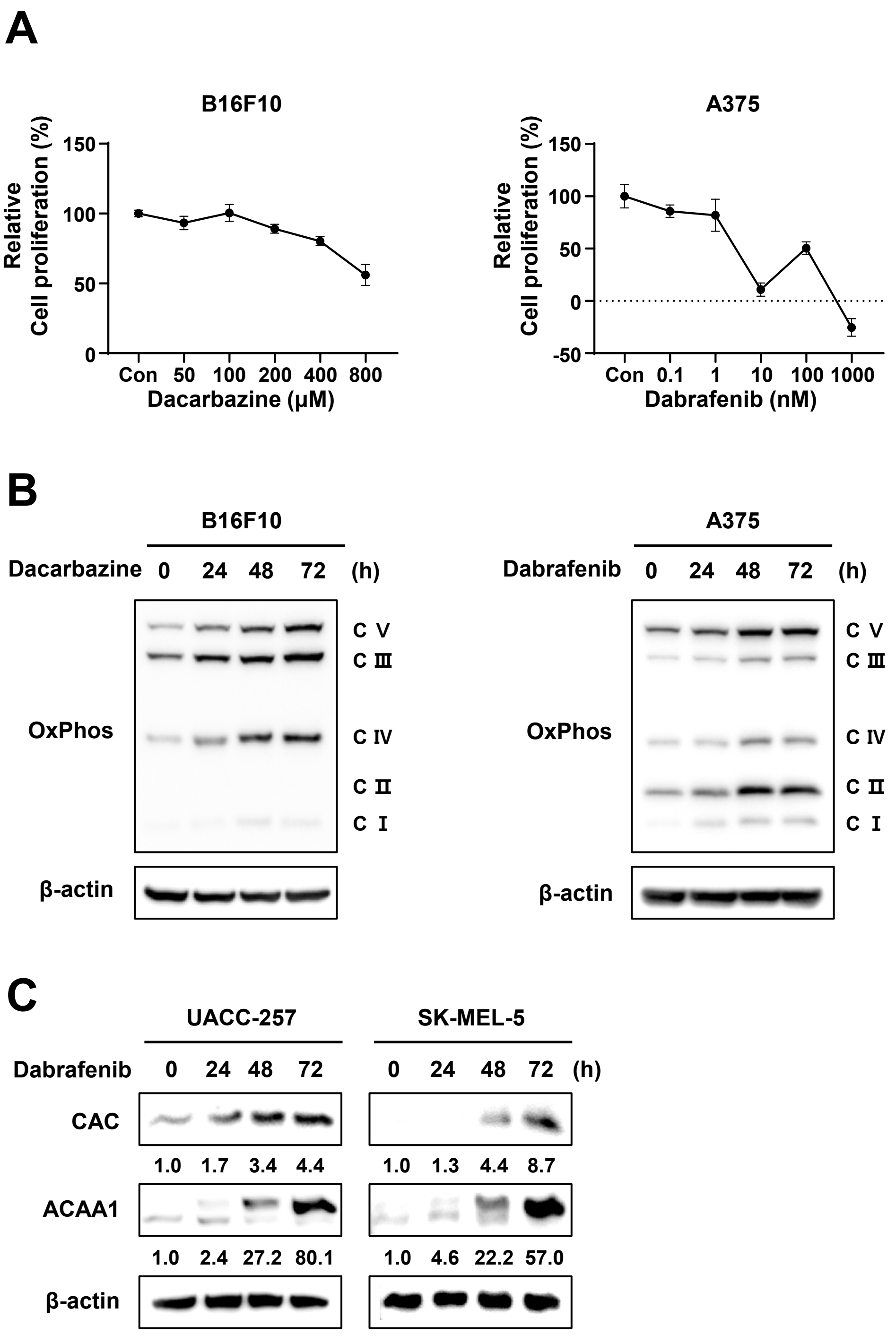

Supplementary Figure S 3. Effects of Dacarbazine and Dabrafenib on cell growth and mitochondrial/ FAO protein expression. (A) Growth-inhibitory effects of Dacarbazine and Dabrafenib in B16F10 and A375 cells. B16F10 and A375 cells were treated with Dacarbazine (Control, 50, 100, 200, 400, and 800 μM) or Dabrafenib (Control, 0.1, 1, 10, 100, and 1000 nM) for 48 h. Cell proliferation was assessed using the SRB assay, and absorbance was measured at 515 nm to determine relative cell proliferation. **(B)** B16F10 cells were treated with Dacarbazine (200 μM), and A375 cells were treated with Dabrafenib (50 nM) and harvested at 0, 24, 48, and 72 h for immunoblotting. The expression of OxPhos complexes was analyzed, and most complexes increased with longer Dacarbazine or Dabrafenib exposure. **(C)** Melanoma cell lines UACC-257 and SK-MEL-5 were treated with Dabrafenib (50 nM) and harvested at 0, 24, 48, and 72 h for immunoblotting. Protein levels of the FAO markers CAC and ACAA1 were examined, showing a progressive increase with longer drug exposure.

Supplementary Figure S4.

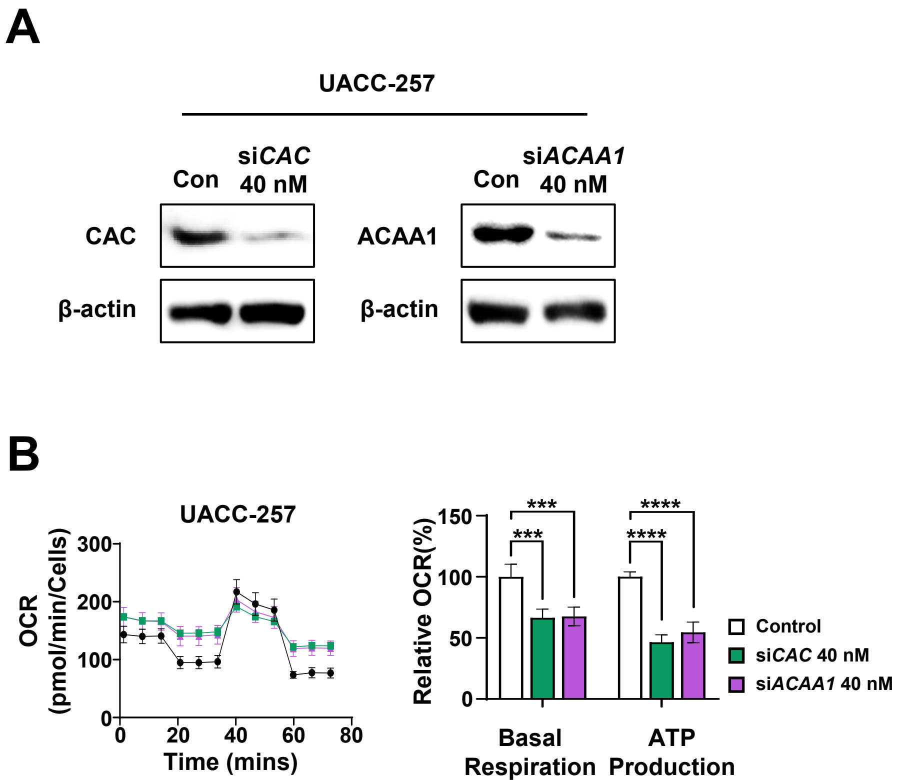

Supplementary Figure S 4. Knockdown of fatty acid oxidation enzymes reduced mitochondrial function in UACC-257 cells. (A) Immunoblotting analysis was performed in UACC-257 cells to confirm the knockdown efficiency of CAC and ACAA1. UACC-257 cells were transfected with si*Scramble* (Control), si*CAC*, or si*ACAA1* (40 nM each) for 72 h. Protein expression of CAC and ACAA1 were reduced compared with the control group. **(B)** Oxygen consumption rate (OCR) analysis was performed using the Seahorse XFe96 analyzer to determine basal respiration and ATP production. After 72 h of siRNA transfection with si*Scramble* (Control), si*CAC*, or si*ACAA1* (40 nM each), data were normalized to SRB assay quantification. Both basal respiration and ATP production were significantly decreased upon *CAC* or *ACAA1* knockdown compared with the control group.

Supplementary Figure S5.

A

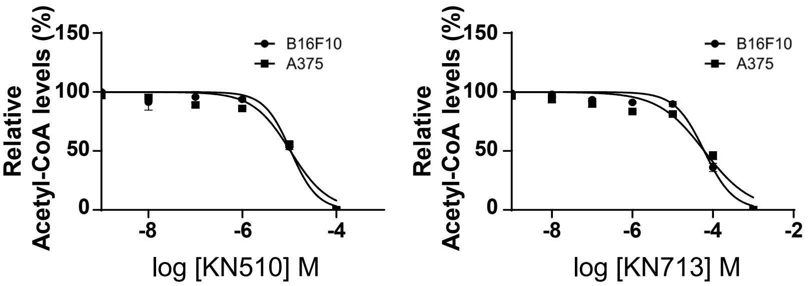

Acetyl-CoA	B16F10	A375
**KN510 (μM)**	10.7	10.5
**KN713 (μM)**	60.4	60.6

B

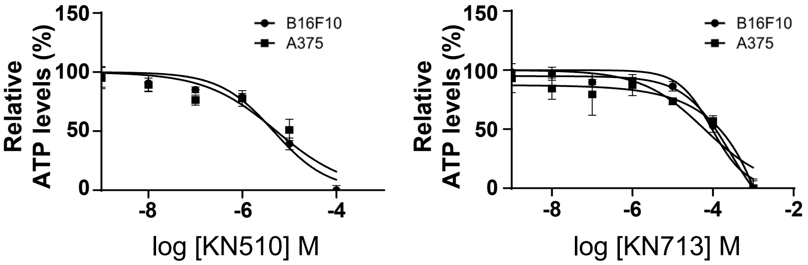

ATP	B16F10	A375
**KN510 (μM)**	4.6	5.3
**KN713 (μM)**	94.5	61.2

Supplementary Figure S5. FAO inhibitors suppressed the growth of melanoma cells. KN510 is an omeprazole that inhibits CAC by targeting the active-site cysteine with strong inhibition[39,40]. KN713 is a trimetazidine that competitively inhibits ACAA, acting on a wide range of substrates from short-chain to long-chain fatty acids[41]. IC_{50} is generally used to evaluate how effectively a drug or compound inhibits cancer cell viability[42]. However, because KN510 and KN713 are non-cytotoxic drugs, the in vitro proliferation assay was redefined. **(A)** IC_{50} was defined as the concentration that inhibits acetyl-CoA production by 50% in cells. The IC_{50} values of KN510 were 10.7 μM and 10.5 μM, and those of KN713 were 60.4 μM and 60.6 μM in B16F10 and A375 cells, respectively. **(B)** IC_{50} was also defined as the concentration that reduces ATP production by 50% in cells. The IC_{50} values of KN510 were 4.6 μM and 5.3 μM, and those of KN713 were 94.5 μM and 61.2 μM in B16F10 and A375 cells, respectively.
